# Dual Roles of Palladin Protein in *In Vitro* Myogenesis: Inhibition of Early Induction but Promotion of Myotube Maturation

**DOI:** 10.1371/journal.pone.0124762

**Published:** 2015-04-14

**Authors:** Ngoc-Uyen-Nhi Nguyen, Hao-Ven Wang

**Affiliations:** 1 Department of Life Sciences, National Cheng Kung University, Tainan, Taiwan; 2 University Center for Bioscience and Biotechnology, National Cheng Kung University, Tainan, Taiwan; 3 Center for Cell Dynamics, National Cheng Kung University, Tainan, Taiwan; Institut de Myologie, FRANCE

## Abstract

Palladin is a microfilament-associated phosphoprotein whose function in skeletal muscle has rarely been studied. Therefore, we investigate whether myogenesis is influenced by the depletion of palladin expression known to interfere with the actin cytoskeleton dynamic required for skeletal muscle differentiation. The inhibition of palladin in C2C12 myoblasts leads to precocious myogenic differentiation with a concomitant reduction in cell apoptosis. This premature myogenesis is caused, in part, by an accelerated induction of p21, myogenin, and myosin heavy chain, suggesting that palladin acts as a negative regulator in early differentiation phases. Paradoxically, palladin-knockdown myoblasts are unable to differentiate terminally, despite their ability to perform some initial steps of differentiation. Cells with attenuated palladin expression form thinner myotubes with fewer myonuclei compared to those of the control. It is noteworthy that a negative regulator of myogenesis, myostatin, is activated in palladin-deficient myotubes, suggesting the palladin-mediated impairment of late-stage myogenesis. Additionally, overexpression of 140-kDa palladin inhibits myoblast differentiation while 200-kDa and 90-kDa palladin-overexpressed cells display an enhanced differentiation rate. Together, our data suggest that palladin might have both positive and negative roles in maintaining the proper skeletal myogenic differentiation *in vitro*.

## Introduction

Skeletal muscle differentiation is a highly ordered multiphase process from myoblast proliferation, to fusion, to myotube differentiation [1–3]. The murine myoblast cell line C2C12 can faithfully mimic skeletal muscle differentiation *in vitro* and serves as an excellent cell model system for investigating the molecular basis of myogenic differentiation [4, 5]. At the onset of differentiation, myoblasts undergo a period of proliferation, and subsequently start to express Myf5 and MyoD, which trigger myoblasts to enter the differentiation program by binding to the E-box CANNTG consensus sequence of the promoter of muscle-specific genes and activate their transcription, including that of transcription factor myogenin [6]. The expression of myogenin facilitates cell fusion and commits myoblasts to withdraw from the cell cycle [7]. The cyclin-dependent kinase inhibitor p21 is upregulated shortly following myogenin expression to prevent phosphorylation of the retinoblastoma protein and is responsible for the inhibition of numerous cyclin-dependent kinases crucial for cell proliferation [8, 9]. Morphologically, myoblasts still appear mononucleated but irreversibly withdraw from the cell cycle. In this phase, a portion of undifferentiated or partly differentiated cells undergoes apoptosis [10]. Mononucleated myoblasts then pair, align, and fuse with adjacent myoblasts to form multinucleated myotubes with centralized nuclei and express terminal differentiation markers and structural proteins such as muscle creatine kinase, sarcomeric α-actinin, and myosin heavy chain (MyHC). In late myogenic differentiation events, myotubes undergo further maturation to generate functional muscle cells, as evidenced by increases in size and changes in the expression of contractile proteins [7, 11, 12]. The multistep process of skeletal myogenesis necessitates intensive actin cytoskeleton remodeling, including myoblast locomotion, elongation, adhesion, fusion, positioning of myonuclei, and bundling of actin filaments to form myofibrils [13]. The sub-cellular coordination of the cytoskeleton and its regulatory, scaffolding, and cytoskeletal cross-linking proteins are responsible for reorganizations and maintaining the normal actin cytoskeleton during myogenesis [14–16].

The actin-organizing protein palladin has been shown to interact with actin and numerous actin-associated proteins that are required for organizing the actin-cytoskeleton to control cell shape, migration, invasion, and development [17–23]. Palladin, whose name describes its function, a scaffold of cells, was first identified and named by Dr. Otey and Dr. Carpén [18, 24]. Palladin is expressed in both muscle and non-muscle cells and tissues, and is present in focal adhesions, membrane ruffles, podosomes [25], the leading edge of astrocytes [26], neurite outgrowths and growth cones [27], and wound granulation tissue [28]. In vertebrates, several palladin isoforms are transcribed from a single gene through alternative splicing [29–31]. Three canonical isoforms of palladin have been characterized, with molecular weights of 200, 140, and 90-kDa, respectively [17, 18]. The largest isoform, 200-kDa palladin, is mainly expressed in the adult heart, skeletal muscle, and testes [31]. The 140-kDa isoform abundantly appears in cardiac muscle and tissues rich in smooth muscle [31]. The 90-kDa isoform, the most common one, is ubiquitously expressed in a variety of cells [31]. Palladin has been reported to control many cellular viability functions, including differentiation processes in myofibroblasts [28] and smooth muscle cell differentiation [32]. However, its role in skeletal muscle differentiation is less understood. Understanding the role of this scaffolding protein in differentiation may be useful for regeneration studies.

Myostatin is a member of the TGF-β superfamily and acts as a negative regulator of muscle growth [33]. Myostatin is expressed almost exclusively in skeletal muscle cells. Overexpression of myostatin suppresses the formation of multinucleated myotubes [34, 35]. Furthermore, mice that exhibited myostatin overexpression had significantly lower muscle mass, fiber diameter, and myonuclei number [36]. Moreover, clinical studies on humans demonstrated a relationship between myostatin expression and muscle atrophy [37], obesity [38], and diabetes [39]. Reciprocally, silencing of myostatin results in muscle hypertrophy and hyperplasia. In addition, natural myostatin mutations that disrupt myostatin activity lead to gains in muscle weight in cattle. Thus, myostatin is an important regulator of skeletal muscle size and growth.

We report here that palladin might play dual roles in the control of myogenesis *in vitro*. In the current study, palladin is knocked down using the short hairpin RNA (shRNA) approach. The shRNA-based depletion of palladin in myoblasts promotes cell cycle withdrawal and early myogenic differentiation after differentiation stimuli, as assessed by the expression of p21, myogenin, MyHC, and MEF2C, and a cell fusion index. However, palladin-deficient myotubes are thinner than those in the control group. This morphology might result from an increase in myostatin expression in palladin-depleted cells. Moreover, palladin depletion inhibits apoptosis *via* the reduction of Caspase-7 activity. Overall, the data sheds light on a newly discovered regulatory pathway in which palladin controls skeletal myogenesis *in vitro*.

## Materials and Methods

### Antibodies and reagents

C2C12 cells were obtained from the Bioresource Collection and Research Center, Taiwan. Oligonucleotides were purchased from BD Biosciences, Taiwan. Dulbecco’s modified Eagle’s medium (DMEM) was obtained from HyClone, USA. Penicillin/Streptomycin was purchased from Nutricell, USA. Fetal bovine and horse sera were purchased from Gibco, USA. Plastic culture dishes and transwell chambers were purchased from Corning, USA. Migration inserts were obtained from idibi, Germany.

The following reagents were used: Caspase-Glo 3/7 assay (Promega, USA), caspDEFINE Caspase-7 Immunoassay Kit (Genetex, USA), Transcriptor First Strand cDNA Synthesis Kit (Roche, USA), 3-(4,5-dimethyl thiazol-2-yl-)-2,5-diphenyl tetrazolium bromide (MTT) (Sigma-Aldrich, USA), crystal violet solution (Sigma-Aldrich), puromycin (Calbiochem, CA), G418 (Sigma-Aldrich), polybrene (Sigma-Aldrich), Lipofectamine 3000 (Invitrogen, USA), TRIzol reagent (Invitrogen), SYBR Green Master Mix Reagent (Applied Biosystems, USA), Bio-Rad Protein Assay Dye Reagent (Bio-Rad Laboratories, USA), a protease and phosphatase inhibitor cocktail (Sigma-Aldrich), RIPA buffer (Cell Signaling Technology, USA), ECL immunoblotting kit (GE Healthcare, USA), DAPI (Invitrogen), and mounting medium (Invitrogen).

The following primary antibodies were used: mouse anti-palladin (1:2000, Novus, USA), mouse anti-gapdh (1:5000, Novus), mouse anti-myogenin (1:500, Genetex, USA), mouse anti-MyHC (1:2000, Genetex), rabbit anti-palladin (1:3000, Proteintech, USA), rabbit anti-tubulin (1:5000, Santa Cruz Biotechnology, USA), rabbit anti-myosin light chain (1:2000, Genetex), and rat anti-myostatin (1:1000, Genetex). Immunofluorescence secondary antibodies and TRITC-phalloidin were obtained from Life Technologies, USA and used at a ratio of 1:400. Horseradish peroxidase-conjugated secondary antibodies were purchased from Jackson ImmunoResearch, USA, and used at a ratio of 1:5000.

### Cell culture and transfection

C2C12 skeletal muscle cell lines (control and transfectants) were propagated as myoblasts in growth medium (GM) containing DMEM supplemented with 10% fetal bovine serum at 37°C in a humidified atmosphere of air and 5% CO_2_. For differentiation into myotubes, confluent C2C12 myoblasts were switched to a differentiation medium (DM) containing DMEM supplemented with 2% horse serum. The myotubes began to form 2–4 days post-differentiation. Every 48 h, the myotubes were fed with fresh DM.

For overexpression of palladin in C2C12 cells, full-length murine palladin isoforms were cloned into plasmid pEGFP-N1 (Clontech, Japan). The following plasmid expression vectors were used: EGFP-90-kDa palladin, EGFP-140-kDa palladin, EGFP-200-kDa palladin, and EGFP alone. C2C12 myoblasts were transfected with 5 μg of the linear plasmid and Lipofectamine 3000 reagent according to the manufacturer’s instructions. At 48 h post-transfection, the transfected-myoblasts were selected under 2 mg/mL of geneticin (G418) for an additional 5 days. Selected cells were seeded in equal numbers for 1 day in GM and incubated for an additional 5 days in DM.

### shRNA infection and selection

To establish stable transfectants, C2C12 myoblasts were separately infected with the appropriate pLKO.1 lentiviral vectors containing scrambled or palladin shRNAs as recommended by the National RNAi Core Facility, Academia Sinica, Taiwan (RNAiCore). Lentivirus particles were purchased from RNAiCore. Briefly, proliferating C2C12 cells at 60% confluence were infected twice overnight with 0.5 mL of viral supernatant containing 8 μg/mL polybrene in serum-free/antibiotic-free DMEM. Fresh GM containing 2.5 μg/mL puromycin was added the next day. Cell populations, which survived in a continuous presence of puromycin after two weeks, were either harvested (as stable cells) and stored or used immediately. Palladin knockdown efficiency was checked using both real-time polymerase chain reaction (qPCR) analysis and western blots. The murine palladin shRNA sequences were: shpalladin-1; 5′-CCGGGCTAACCTATGAGGAAAGAATCTCGAGATTCTTTCCTCATAGGTTAGCTTTTT -3′, shpalladin-2; 5′-CCGGAGCCAAAGATCTATTGGTTTACTCGAGTAAACCAATAGATCTTTGGCTTTTTTG-3′. The scrambled shRNA lentivirus with a hairpin sequence targeted to firefly luciferase was used as a control shScrambled; 5′ CCGGCTTCGAAATGTCCGTTCGGTTCTCGAGAACCGAACGGACATTTCGAAGTTTTTG-3′. Hereafter, these pools are referred to as shPalld-1, shPalld-2, and shLuc, respectively.

### Measurement of cell vitality (MTT assay)

The MTT assay was used to examine the changes in cellular viability. C2C12 cells were seeded in equal numbers in 24-well plates. At the indicated time points, the medium was aspirated, and the cells were incubated with fresh medium containing 0.2 mg/mL MTT for an additional 4 h. Following culture medium aspiration, the resulting formazan crystals were dissolved in 100 mL of dimethyl sulfoxide. The absorbance was assessed using a microplate reader (Tecan, Switzerland) at a test wavelength of 570 nm (reference wavelength: 630 nm). The quantity of formazan product in association with the intensity of absorbance was directly proportional to the number of cultured living cells. The data were obtained from three independent assays.

### Caspase 3/7 cell apoptosis assay

Cells were grown in 96-well plates until 70–80% confluence and shifted to DM at the indicated time. Apoptotic cells were detected using Caspase-Glo 3/7 assay according to the manufacturer’s instructions. At 0, 24, and 48 h after differentiation induction, 100 μL of Caspase-Glo 3/7 reagent was added to each well. The cells were then incubated for 30 min at room temperature. Subsequently, the luminescence of each sample was measured using a Tecan Infinite F200 PRO enzyme-linked immunosorbent assay (ELISA) reader.

### Caspase-7 immunofluorescence apoptosis assay

Cells were seeded in 60-mm culture dishes, induced for differentiation and harvested at the indicated time points. Cell lysates were prepared, and 200 μg of proteins was used for Caspase-7 activity assays using a caspDEFINE Caspase-7 Immunoassay Kit. Samples were read at 450 nm in a fluorescence microtiter plate reader. The fold increase in Caspase-7 activity was determined by comparison with the level in control cells.

### Western blots

Protein was extracted from C2C12 cell cultures using RIPA buffer with a protease and phosphatase inhibitor cocktail. The protein concentration was determined using the Bradford method. Equal amounts (30 μg) of cell protein extracts were resolved on SDS-PAGE. Resolved proteins were electrophoretically transferred to nitrocellulose membranes, and subjected to immunoblotting. Membranes were blocked with 5% skim milk in Phosphate buffered saline (PBS) for 1 h. Membranes were incubated with primary antibodies at 4°C overnight and secondary antibodies for 1 h at room temperature.

### RNA isolation and quantitative real-time PCR (qPCR) analysis

Total RNA from cultured cells was isolated by TRIzol extraction according to the manufacturer’s instructions. cDNA was synthesized from 1 μg of total RNA with the use of a Transcriptor First Strand cDNA Synthesis Kit (Roche). For qPCR reaction, synthesized cDNA and SYBR Green Master Mix Reagent were run on a StepOnePlus Real-Time PCR system (Applied Biosystems, Life Technologies) with 250 nM primers. All reactions were run in triplicate, and PCR product size was verified using melting curve analysis. The relative expression of mRNA was determined after normalization to gapdh levels using the ΔΔ*CT* method. The mRNA level of scrambled knockdown cells on day 0 was used as a reference. All primer sequences are available on request (S1 Table).

### Microscopy and myogenic index

Approximately 5×10^5^ myoblasts transfected with scrambled shRNA or palladin-specific shRNAs were seeded in 10 cm plates and photographed daily using a Nikon digital camera (Nikon, Japan) with a 10× objective lens.

Cell differentiation was assessed by quantifying the number of MyHC-positive cells and measuring the fusion index. Palladin-knockdown and control cells were grown in equal numbers on 0.2% gelatin-coated glass coverslips to 80% confluence in GM and then shifted to DM. Cells were harvested at the indicated time points, fixed with 4% paraformaldehyde for 15 min, and permeabilized with 0.2% (v/v) Triton X-100 for 15 min. After 1 h of blocking in 1% (w/v) bovine serum albumin in PBS, samples were probed with murine MyHC and rabbit palladin antibodies and the proper fluoresceine-conjugated antibodies. For F-actin visualization, cells were stained with TRITC-conjugated phalloidin for 30 min at room temperature. Nuclei were counterstained with DAPI dye for 1 min at room temperature. Photographs were taken at 10× magnification with an Eclipse T*i* epifluorescence microscope (Nikon). The total number of MyHC-positive cells was calculated. The fusion index was determined as the percentage of the average number of nuclei in MyHC-positive cells with at least three nuclei to the total nuclei [40]. At least 1000 cells were counted for each condition, and four separate trials were performed independently. Values were analyzed, and histograms were generated using GraphPad Prism version 5.0 (GraphPad Software, La Jolla, CA).

### Cell size determination

Following the time course experiment, immunofluorescence microscope images were taken, and the myotube diameter was assessed using ImageJ software (National Institutes of Health, Frederick, MD, USA). To analyze diameters and lengths, the 50 largest myotubes for each cell line were measured, and the mean ± standard deviation (SD) values were calculated. Myotube diameters and lengths were determined as the average values from three independent measurements.

### Scratch wound-healing assay

C2C12 cells were seeded on separate inserts according to the manufacturer’s instructions (ibidi, Germany) for 24 h. The inserts were then carefully removed to release the wounds. Cells that converged to close the wound were photographed with a 10× objective at two preselected time points (0 and 12 h) using an optical microscope (Nikon). The wound closure areas were evaluated using TScrash analysis software [41] and plotted for statistical analysis. The experiment was independently performed at least three times.

### Transwell migration assay

Transwell migration was tested using 24-well format 8.0-μm pore size transwell systems as previously described [42]. 2 × 10^4^ cells were seeded in the upper chamber, and DM was added into the lower chamber of the transwell plate. Cells were allowed to migrate for 12 h. Non-migrated cells on the top side of the filter were gently wiped with cotton swabs. The cells that migrated through the filter membrane were fixed with 4% formaldehyde for 15 min, washed with PBS, stained with 0.05% crystal violet solution for 30 min, and rinsed with PBS again. The filters were cut out from the transwell inserts, mounted onto glass slides with mounting medium, and photographed under a Nikon light microscope. The migrated cells in three random fields of view were determined using ImageJ software. Each experiment was performed at least in triplicate.

### Statistical analysis

Results for individual cell experiments were replicated in at least three independent experiments (n≥3), each performed with triplicate samples and are presented as the mean ± SD. Data were analyzed using two-tailed Student's *t-*test for two-group comparisons, and two-way repeated-measures analysis of variance (ANOVA) with Bonferroni post-tests for multiple group comparisons at various time points. GraphPad Prism version 5.0 was used for analysis. The values of p≤0.05 (*), p≤0.01 (**), and p≤0.001 (***) were considered statistically significant.

## Results

### shRNA-mediated knockdown of palladin accelerates the onset of C2C12 myoblast differentiation

**Fig 1:**
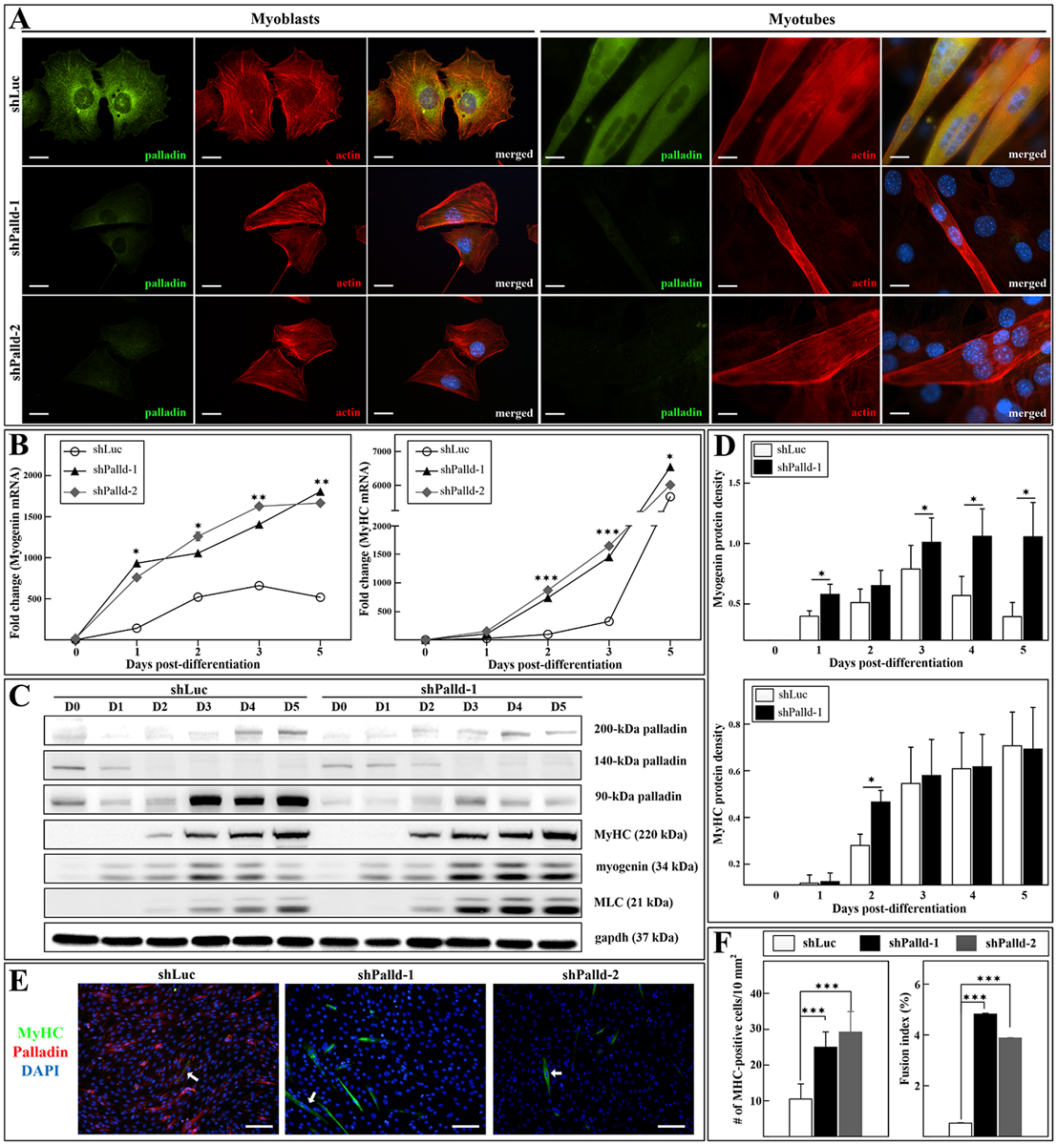
shPalladin-harboring myoblasts displayed propulsive myogenic differentiation via precocious expression of myogenic differentiation markers. C2C12 cells were transfected with shRNA targeting palladin or nontargeting shRNA as a control. Stable transfectants were submitted to a differentiation medium and followed for 5 days in culture (D0 to D5). Myogenic differentiation was assessed at the indicated time points using RNA/protein analysis and immunofluorescence staining. **(A)** Immunofluorescence images of stable transfectants labeled with palladin antibody (green) and F-actin (red). Blue is DAPI-stained nuclei. Scale bar is 10 μm. **(B)** qPCR was performed to assess the expression of differentiation markers myogenin (left) and MyHC (right). Note that the expression of myogenin and MyHC in palladin-knockdown cells is higher than that of control cells. **(C)** Western blots analysis of C2C12 cells labeled with a monoclonal antibody against 90-kDa palladin or a polyclonal antibody against 140- and 200-kDa palladin, MyHC, myogenin, and MLC. The blots clearly show the decrease in palladin expression concomitant with the elevation of differentiation markers. **(D)** Expression levels of myogenin (top) and MyHC (bottom) protein were quantified relative to gapdh using densitometric analysis. **(E)** Immunofluorescence of representative image fields of scrambled and palladin-knockdown cells after two days in DM, labeled for palladin (red), MyHC (green), and DNA (blue). Arrows indicate the MyHC-positive cells. Scale bar is 100 μm. **(F)** Differentiation was quantified by the number of MyHC-positive cells (left) and the fusion index (right) at day 2 of differentiation. The fusion index was defined as the number of nuclei in myotubes / total number of myonuclei (a myotube is defined as having at least three nuclei). Results are shown as mean ± SD of data from at least three independent determinations. * indicates statistically significant difference from control cells, * *p<*0.05, ** *p<*0.01, *** *p<*0.001 by two-way ANOVA (B, *n = 3*) or Student’s *t*-test (D, F, *n = 4*).

To elucidate the functional roles of palladin in myogenic differentiation, we created C2C12 stable cell lines deficient in palladin or scrambled controls using shRNAs. C2C12 myoblasts were infected with lentivirus expressing either shPalladin or shLuc. Firstly, we examined whether shLuc-infected cells had undergone the proper differentiation process. As shown in S1 and S2 Figs, under differentiation induction, shLuc-infected cells fused to form multinucleated myotubes, expressed sarcometric proteins (arrow), and assembled myofibrils structures (arrowheads). Thus, they can serve as control cells. The efficacy of lentiviral-mediated knockdown of palladin was confirmed. Knockdown cells significantly reduced palladin mRNA content (S3 Fig). As anticipated, immunofluorescence results showed that palladin (green) was knocked down in both shPalladin-harboring myoblasts and myotubes (Fig 1A). Consistently, palladin-knockdown cells also displayed a significant decrease in palladin protein levels (Fig 1C and S4 Fig). The knockdown effect on myogenesis was firstly quantified by measuring the expression of myogenin and MyHC. Interestingly, knockdown cells prematurely increased the levels of myogenin and MyHC mRNA (Fig 1B), suggesting an accelerated differentiation in C2C12 when palladin is knocked down. Western blots analysis also revealed an earlier expression of MyHC protein (on day 2 of differentiation) in palladin-depleted cells compared to that in control cells (Fig 1C and 1D, S4 Fig). Similarly, protein levels of myogenin also showed a trend of higher levels starting from day 3 of differentiation (Fig 1C and 1D). In order to provide a dynamic analysis of myoblast differentiation into multinucleated myotubes, the myogenic index was assessed by counting MyHC-positive cells (Fig 1E, arrows). Consistently, both the number of MyHC-positive cells and the fusion index significantly increased at day 2 after switching to DM in cells transfected with shPalladin (Fig 1F). These results indicate potentially elevated skeletal myogenesis in palladin-depleted cells.

### Inhibition of palladin induces expression of p21 and inhibits serum withdrawal-induced apoptosis during myoblast differentiation dependently on Caspase-7 activity

**Fig 2:**
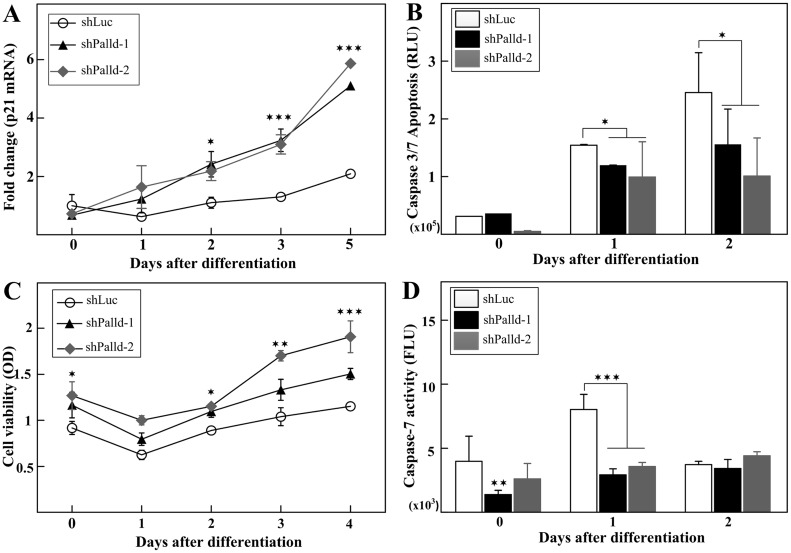
Palladin-depleted myoblasts induce p21 expression and decrease Caspase7-dependent apoptosis. **(A)** qPCR analysis of p21 was performed to assess the expression of cell cycle withdraw marker in control and palladin-knockdown cells. **(B)** Time course of the apoptosis quantification in palladin-knockdown and control cells measured using Caspase-Glo 3/7 luminescence assay. **(C)** Time course of palladin-knockdown and control cells survival measured using MTT assay. **(D)** Time course of the apoptosis quantification in palladin-knockdown and control cells measured using Caspase-7 immunofluorescence assay. All data represent at least three independent experiments. Values are presented as the means ± SD. * indicates statistically significant difference from control cells, * *p<*0.05, ** *p<*0.01, *** *p<*0.001 by two-way ANOVA (A, C) or Student’s *t*-test (B, D).

The presence of differentiation markers is associated with an irreversible withdrawal of myoblast cells from the cell cycle upon serum starvation. Thus, the expression of cyclin-dependent kinase inhibitor p21 (Waf1, Cip1) was examined as a marker of terminal cell cycle arrest in knockdown cells. As shown in Fig 2A, depletion of palladin significantly raised the level of p21 mRNA. In addition, the qPCR approach also showed that the abundance of MEF2C gradually increased during differentiation in palladin-knockdown cells (S5 Fig). These data reveal that palladin inhibition leads to the coupling of cell cycle withdrawal with the induction of muscle-specific genes, and thus promotes the initial steps of myoblast differentiation.

During myoblast differentiation, a portion of cells must be removed through apoptosis. Given that p21 can protect cells from apoptosis in response to growth factor deprivation, we next assayed whether the up-regulation of p21 expression in palladin-depleted myoblasts would render cells more resistant to apoptosis. Cells with palladin inhibition displayed a significant decrease in their apoptosis rate compared with that of the control cells after differentiation induction (Fig 2B). Consistent with reducing apoptosis, cell survival in palladin-knockdown cells increased compared to that of the control as measured by the MTT assay (Fig 2C). Moreover, Caspase-7 assays showed that the depletion of palladin decreased apoptosis through the reduction of Caspase-7 activity (Fig 2D). Hence, these results suggest that the attenuation of palladin in myoblasts decreases the Caspase-7-dependent apoptosis in C2C12 cells.

### Loss of palladin expression results in inhibition of mature myofiber formation

**Fig 3:**
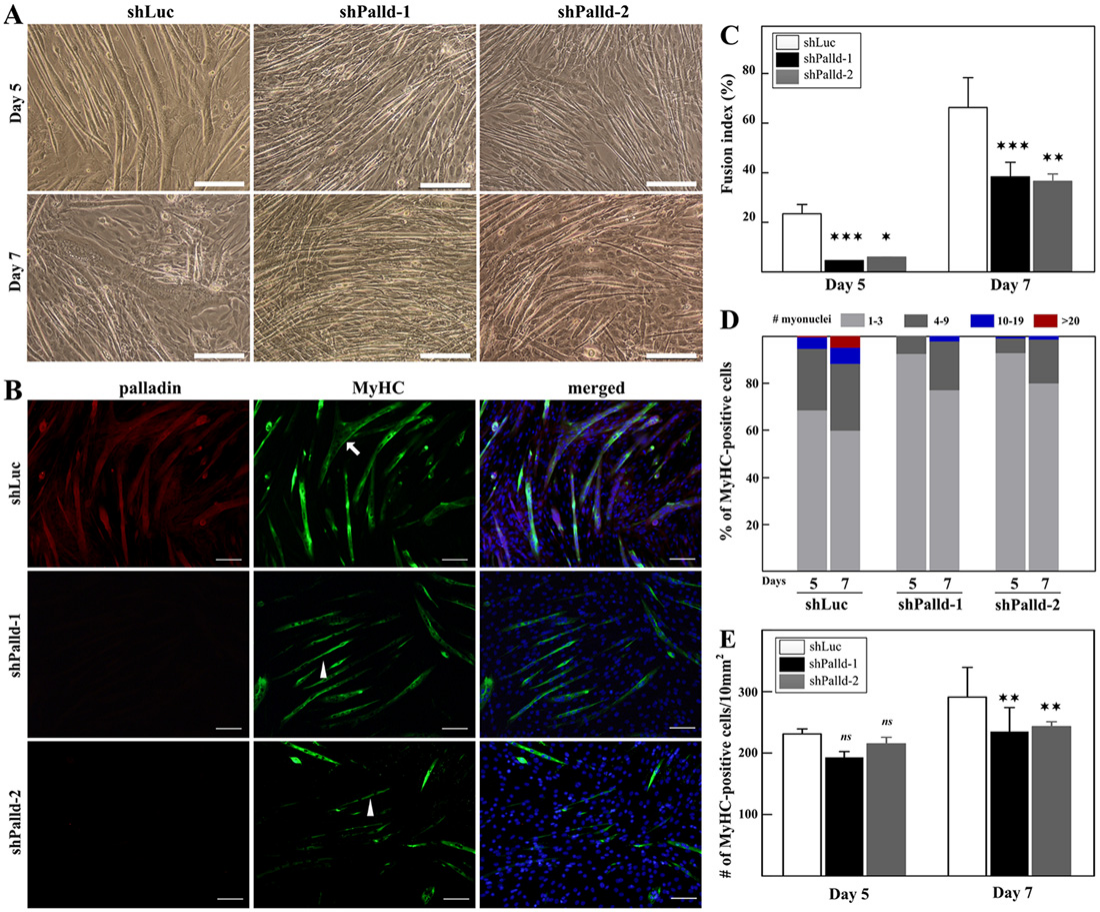
Effect of palladin depletion on terminal differentiation of C2C12 cells. **(A)** Phase contrast images of stable transfectants at late stages of differentiation. Scale bar is 50 μm. **(B)** Representative immunofluorescence images of stable transfectants at day 5 of differentiation. Cells were labeled with palladin (red), MyHC (green), and DAPI (blue). Scale bar is 100 μm. **(C)** Fusion index analysis of stable transfectants at day 5 (left) and day 7 (right) of differentiation. A minimum of 4,000 nuclei were counted from random fields of each cell line. Note that palladin depletion resulted in a decrease of the fusion index at the late stage of differentiation. **(D)** Quantification of multinucleated myotubes throughout the course of differentiation. **(E)** Quantification of the number of MyHC-positive cells in stable transfectants. All error bars indicate the means ± SD of at least four independent experiments. * indicates statistically significant difference from control cells, * *p<*0.05, ** *p<*0.01, *** *p<*0.001 by Student’s *t*-test. *ns* = not significant.

We next investigated the terminal differentiation by analyzing both the morphology and differentiation index on days 5 and 7. As aforementioned, shPalladin-depleted cells formed more nascent myotubes (fewer than 4 myonuclei) than did control cells at day 2 of differentiation. Based on this earlier differentiation, it was speculated that palladin-deficient cells would form more mature myotubes than would the control group. Intriguingly, knockdown cells later displayed a reversal effect on the formation of mature myotubes. Palladin-knockdown myotubes appeared smaller than control cells, both under phase microscopy (Fig 3A) and fluorescence staining (Fig 3B). Indeed, knockdown cells had a decreased cell fusion index relative to that of control cells (Fig 3C). Consistently, palladin-repressed myotubes contained fewer myonuclei than those of control myotubes. A dynamic analysis of these data is shown in Fig 3D. Additionally, knockdown cells formed fewer MyHC-positive cells at the late stage of differentiation (Fig 3E, day 7). Thus, it seems that palladin-depleted cells are unable to form mature myotubes.

### Palladin depletion-induced expression of myostatin in C2C12 cells caused thinner myotubes

**Fig 4:**
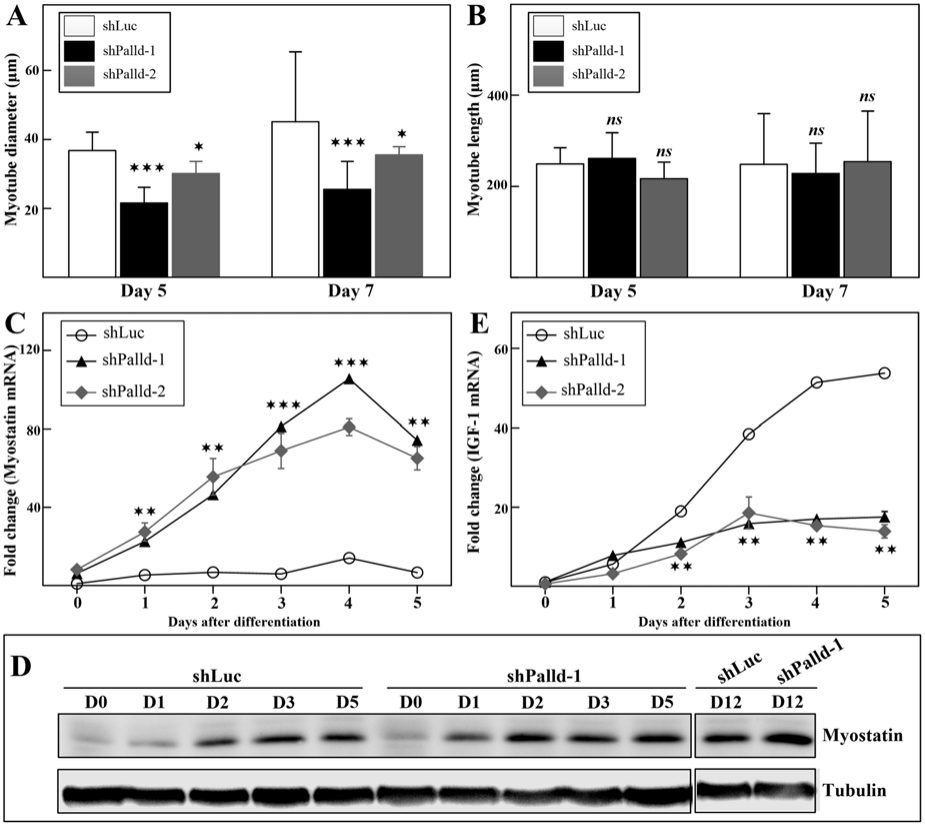
Loss of palladin expression results in increased myostatin activity. Quantification of myotube **(A)** width and **(B)** length of stable transfectants at late-stage differentiation (day 5 and day 7). qPCR was performed to assess the expression of **(C)** myostatin and **(E)** IGF-1 during myoblast differentiation. **(D)** Western blots analysis of palladin-knockdown and control cells labeled with an antibody against active myostatin. All error bars indicate the means ± SD of at least three independent determinations. * indicates statistically significant difference from control cells,* p<0.05, ** p<0.01, *** p<0.001 by Student’s t-test (A, B) or two-way ANOVA (C, E). ns = not significant.

Given that smaller myotubes were formed in knockdown cells, the myotube diameter was measured to discriminate between knockdown and control myotubes. As observed, multinucleated myotubes from palladin-deficient C2C12 were thinner than those formed from control cells (Fig 4A). However, the average myotube length was almost unaffected (Fig 4B) when palladin was knocked down. It is known that the diameter of post-differentiated myotubes is decreased concomitantly with elevated myostatin expression [36]. We next examined the activity of myostatin in palladin-depleted myotubes. The mRNA level of myostatin was dramatically increased in palladin-deficient cells during differentiation (Fig 4C). Conceivably, active myostatin protein was also increased in palladin-depleted myotubes, particularly at late-stage myogenesis (day 12) (Fig 4D). IGF-1 attrition is known as one potential cause of the increased myostatin. Therefore, IGF-1 mRNA was also examined. The results showed a significant decrease of IGF-1 mRNA in palladin-deficient myotubes (Fig 4E). Collectively, the results suggest that loss of palladin in C2C12 myoblasts stimulates them to increase myostatin activity.

### Effects of palladin depletion on cell migration

**Fig 5:**
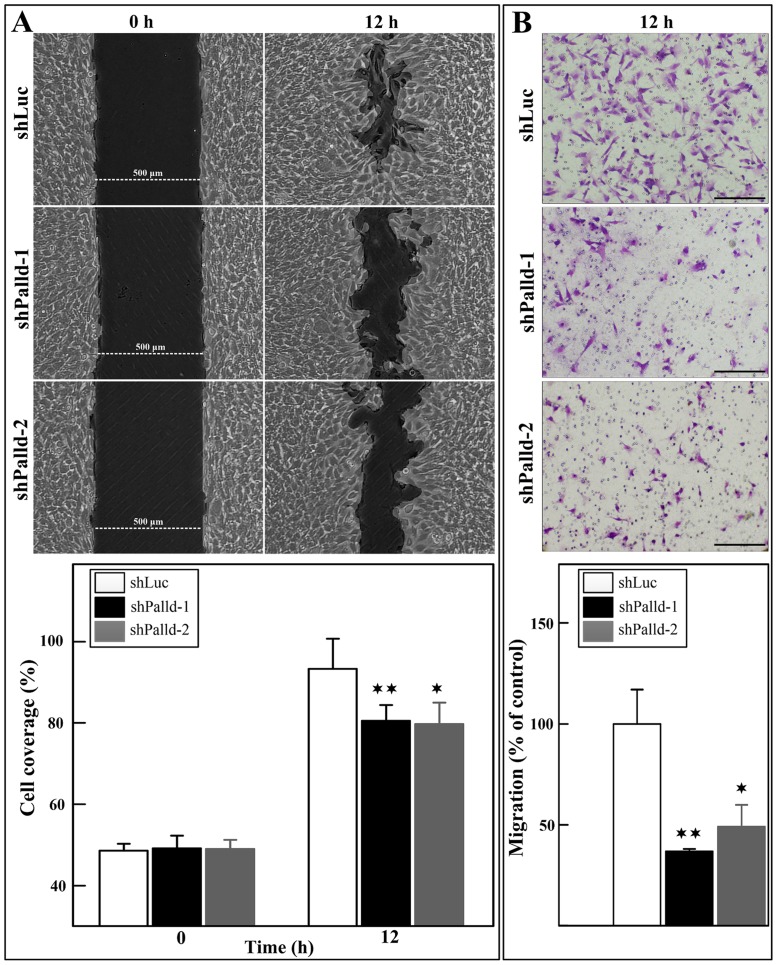
Decrease of cell migration in C2C12 myoblasts by palladin knockdown. **(A)** The wound-healing migration of stable transfectants was recorded with an optical microscope at the indicated time points and subjected to statistical analysis (n = 4 per group). The results are represented as the percentage of cell-covered area (lower panel). **(B)** The stable transfectants were plated onto the upper chamber of transwells (n = 6 per group). After 12 h, myoblasts that migrated to the bottom side of the filter were stained with crystal violet and counted (lower panel). The data were evaluated from at least three independent experiments. Values are presented as the means ± SD. * indicates statistically significant difference from control cells, *p<0.05, **p<0.01 by Student’s t-test. Scale bar is 100 μm.

Given that migration is essential for differentiation, we next investigated whether the motor functions of skeletal muscle are affected in palladin-depleted cells. The scratch wound healing assay results indicated that palladin knockdown caused a decline in the migration index of approximately 13% after 12 h of cell damage induction in comparison to that of control cells (Fig 5A). Furthermore, in the transwell migration assay, nearly 50% fewer of palladin-deficient cells were observed to migrate to the bottom membrane of the transwells when compared to the control cells (Fig 5B). Hence, these results suggest that palladin is essential for the regulation of C2C12 migratory capacity.

### Overexpression of distinct palladin isoforms altered myogenesis *in vitro*

**Fig 6:**
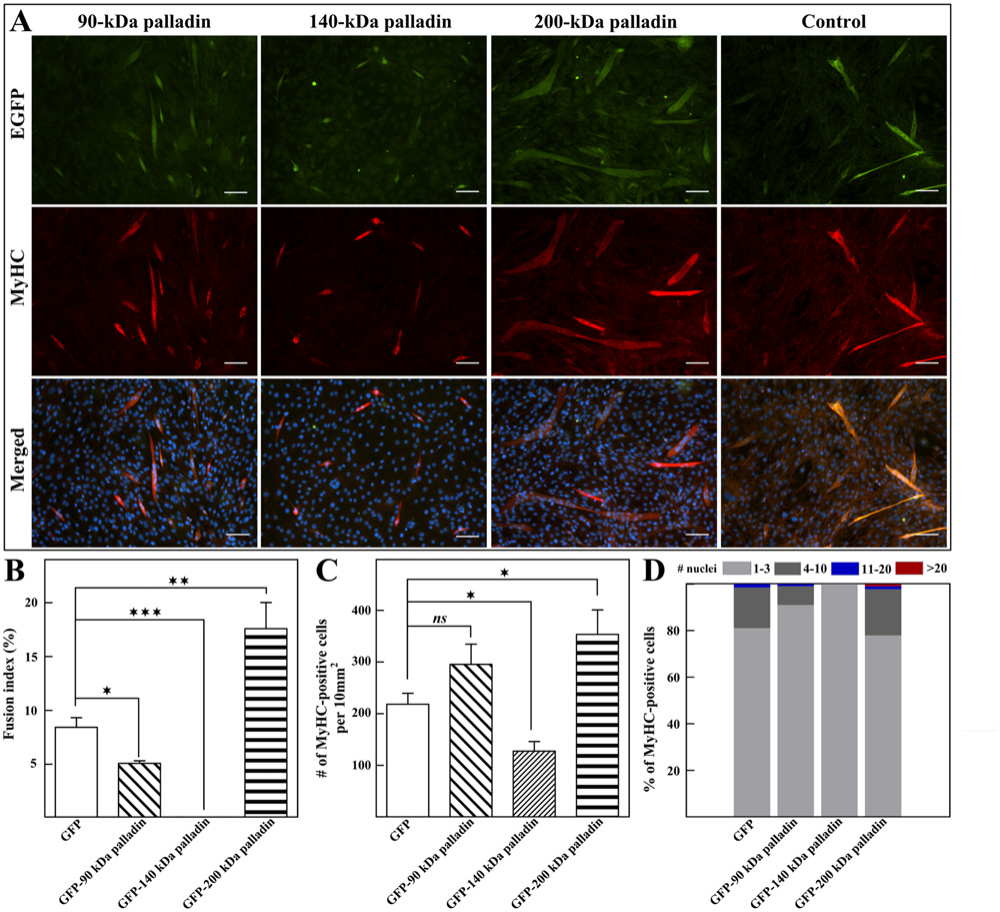
Effect of palladin overexpression on C2C12 differentiation. **(A)** Expression of MyHC (red) by immunofluorescence on C2C12 cells transfected with pEGFP (EGFP) or pEGFP-90-kDa palladin, pEGFP-140-kDa palladin, and pEGFP-200-kDa palladin, and differentiated for 5 days. Nuclei are stained with DAPI. Scale bar is 100 μm. Quantifications of **(B)** fusion index and **(C)** number of MyHC-positive cells of overexpressed cells. All error bars indicate the means ± SD of at least four independent determinations. * indicates statistically significant difference from control cells, * p<0.05, ** p<0.01, *** p<0.001 by Student’s t-test, ns = not significant). **(D)** Quantification of multinucleated myotubes throughout the course of differentiation. Scale bar is 100 μm.

Given that knockdown of palladin resulted in opposite effects in skeletal muscle differentiation, we speculated that different isoforms of palladin are responsible for the separated differentiation stages in myogenesis. We further analyzed the role of palladin isoforms in myogenesis via the transfection of distinct palladin-expressing plasmids into C2C12 cells and analysis of their myogenic index. Immunofluorescence staining with anti-MyHC (in red) was then performed to determine myogenic differentiation on day 5 of differentiation (Fig 6A). Interestingly, the overexpression of 140-kDa palladin remarkably decreased both the fusion index and number of MyHC-positive cells (Fig 6A–6C). More importantly, EGFP-140-kDa palladin-transfected myoblasts failed to form any mature myotubes (Fig 6D). In contrast, both the fusion index and the number of MyHC-positive cells significantly increased when 200-kDa palladin was overexpressed. The overexpression of 90-kDa palladin did not show a significant difference in the number of MyHC-positive cells (Fig 6C). These results suggest the distinct role of palladin isoforms during myogenesis *in vitro*.

## Discussion

**Fig 7:**
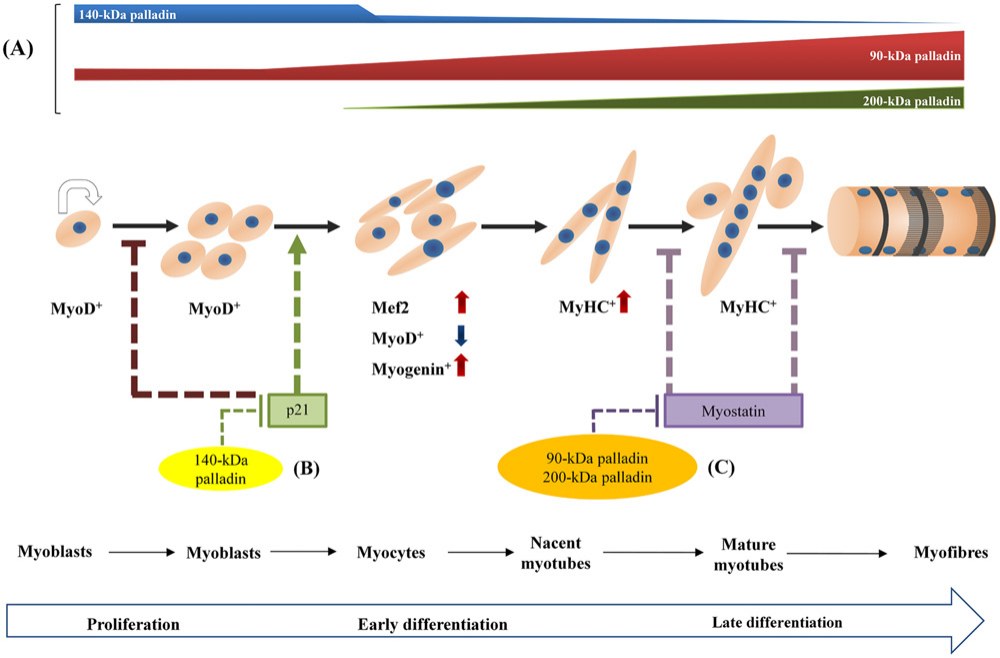
Schematic model outlining how reduced expression of palladin affects skeletal muscle differentiation processes. Schematic diagram showing the involvement of palladin in differentiation of proliferating myoblasts into nascent myotubes and maturation into myofibers. **(A)** Expression of palladin isoforms during myogenesis. 140-kDa palladin is decreased while 90- and 200-kDa palladin are increased. **(B)** In the early stage of differentiation, 140-kDa palladin might promote myoblast proliferation and prevent entry into the differentiation program via the inhibition of p21 activity. Thus, the depletion of palladin promotes cell cycle exit, decreases apoptosis and activates myogenic regulatory factors, especially myogenin and MyHC, allowing myoblasts to start their differentiation program. **(C)** In late differentiation, 90- and 200-kDa palladin might increase the myogenic index through the inhibition of myostatin activity, promoting the formation of mature myotubes. Thus, knockdown of palladin releases myostatin, which results in the formation of thinner myotubes in palladin-depleted cells.

To explore the role of palladin in myogenesis, we first examined its expression profiles in proliferating and differentiating immortalized C2C12 cells. It is well established that the withdrawal of growth factors initiates differentiation and induces expression of the transcription factor myogenin at about day 1–2 and the later structural protein MyHC from day 2–3 after transfer to DM. As shown in Fig 1C, 90-kDa palladin was strongly expressed and increased during *in vitro* differentiation, in parallel with the rise of myogenin and MyHC. 200-kDa palladin also showed increased expression during differentiation, with weaker expression levels than those for 90-kDa palladin (Figs 1C and 7A). These data are consistent with a previous report [31] and indicate the critical role of palladin during *in vitro* skeletal muscle differentiation. In contrast, the expression of 140-kDa palladin decreased in response to serum starvation (Figs 1C and 7A). Immunofluorescence results also showed the periodic, punctate pattern expression of palladin (Fig 1A), as mentioned elsewhere [24, 43].

In the current study, the well-characterized C2C12 mouse myoblast cell culture was used to test the function of actin-associated palladin during skeletal muscle differentiation. The multistep differentiation process of myoblasts entails the coordination of the irreversible exit of proliferating myoblasts from the cell cycle and the expression of muscle-specific genes. At the onset of myoblast differentiation, myogenin mRNA essentially absents at the time of media switch to trigger differentiation, but it was expressed after one day (Fig 1B). Following the expression of the cell cycle inhibitor p21, a negative cell cycle regulator that arrests the cell cycle, proliferating myoblasts exit the cell cycle, acquire an apoptosis-resistant phenotype, and fuse with others. Differentiating myoblasts subsequently activate the expression of motor protein MyHC to promote the formation of multinucleated myotubes. MyHC acts as a determinant for muscle differentiation. Our results showed that shRNA-mediated depletion of palladin encourages the rise of myogenin and MyHC expression (Fig 1B and 1C), and the formation of myotubes in early differentiation phases (Fig 1E and 1F). These data are in line with those of previous studies that indicated that transient depletion of palladin facilitates the myoblast differentiation process [44]. Moreover, loss of palladin also increased the mRNA level of MEF2C (S5 Fig), which belongs to the MEF family of transcription factors that function in the differentiation of myocytes and skeletal muscle growth. In addition, the depletion of palladin also promoted the expression of the p21 gene (Fig 2A) and led serum-starved C2C12 cells to resist apoptosis (Fig 2B). It is well accepted that proliferating myoblasts are far more susceptible to apoptotic cell death compared to those of being differentiated myocytes. When myoblasts no longer continue to generate and have an apoptosis-resistant phenotype, they may enter the cell differentiation program and express muscle-specific genes. Similar to our results, palladin has been reported to promote apoptosis through modulation of the subcellular localization of integrin linked kinase-associated phosphatase in HEK293 cells [45]. Moreover, this apoptosis phenomenon was dependent on Caspase-7 activity (Fig 2D). Collectively, these results underline the function of palladin in the negative regulation of some early stages of myoblast differentiation.

Palladin has been reported as a cytoskeleton-associated protein that interacts with many proteins to promote cell migration [19, 20, 46–48] and actin-cytoskeleton rearrangement. Therefore, it is not surprising that palladin knockdown decreased C2C12 migration (Fig 5). This result is consistent with previous studies, in which transient knockdown of palladin inhibited myoblast migration [44].

Palladin-depleted myoblasts exhibit an increased differentiation rate in the early stage of differentiation. Thus, we speculated that they can fuse and form more differentiated post-mitotic myotubes compared to those of the control group. Contrary to expectations, compared to the control group, myotubes with attenuated palladin expression were thinner and contained fewer myonuclei (Figs 3 and 4A). The inhibition of late myogenesis in knockdown cells was shown here to be associated with the elevation of a TGF-β family member, myostatin (Fig 4C and 4D), and the decrease of IGF-1 mRNA expression (Fig 4E). In vertebrates, skeletal muscle hypertrophy occurs as a result of an increase in skeletal muscle size rather than an increase in fiber number. IGF-1 activity acts as a pro-hypertrophy activator that activates IGF-1/PI3K/Akt signaling and dominantly inhibits the effects of myostatin on muscle growth. An elevated level of myostatin causes a decrease in post-differentiated myotube diameter through the upregulation of the atrophy-related proteins MuRF1 and MAFbx, reduces protein synthesis, and hence prevents muscle growth and regeneration [49, 50]. Furthermore, decrease of palladin mRNA was found in atrophying skeletal muscle response to food deprivation [51]. Therefore, it is reasonable to conclude that palladin acts via the inhibition of myostatin activity during differentiation to prevent the atrophy program. Additionally, myostatin signaling decreases proliferation by stimulating the expression of p21 [52]. Interestingly, overexpression of myostatin protects undifferentiated myoblasts from apoptosis processes [34, 53]. These findings are in line with our apoptosis results (Fig 2B). However, it is interesting to note that the effect of palladin on myostatin expression appears to be specific to myotubes, not myoblasts. It is still unknown whether palladin-mediated myostatin expression is directly controlled by palladin proteins. Nonetheless, elevated myostatin activity in palladin-deficient myoblasts suggests the involvement of palladin in hypertrophy, atrophy, and the cachexia pathways. Furthermore, the exact mechanism for such differentiation effects is unclear, but may be related to the isoform-specific roles of palladin. Palladin has a bimodal expression during myogenesis, increasing in 90- and 200-kDa isoforms, but decreasing in 140-kDa expression. Thus, we speculated that palladin isoforms perform different roles in myogenesis. On the basis of our findings in overexpression experiments, we propose that 140-kDa palladin is essential for the continuation of the cell cycle and proliferation in C2C12 myoblasts. In the absence of 140-kDa palladin, myoblast proliferation and apoptosis are inhibited, and the differentiation process is promoted. On the other hand, 200-kDa palladin is required for late stage differentiation, in part at least, via the fusion process. Thus, we propose the following novel functions of palladin in skeletal myogenesis: 140-kDa palladin acts as a negative regulator to inhibit early differentiation while 90- and 200-kDa palladin promotes late differentiation (Fig 7). However, further studies are needed to confirm this hypothesis.

## Conclusion

In this report, we showed that the shRNA-mediated inhibition of palladin in C2C12 myoblasts stimulates them to exit the cell cycle and express myogenic markers at the early phases of myogenesis, but efficiently retards the formation of multinucleated myotubes via the enhanced activation of myostatin. Palladin-depleted cells may not maintain their ability to form mature myotubes, which is critical for proper myogenesis (Fig 7). Therefore, we propose that palladin negatively controls the commitment of C2C12 cells to differentiation but inhibits myostatin activity to maintain proper terminal differentiation. To our knowledge, this is the first report in the literature to demonstrate the inhibitory and positive effects of palladin on *in vitro* myogenesis.

## Supporting Information

S1 Fig Bright field images of stable transfectants during differentiation process. Arrows indicate the mature myotubes. Arrowheads indicate the thin myotube of palladin-knockdown cell lines.
(TIF)

S2 Fig Immunofluorescence images of scrambled-knockdown cell lines that underwent differentiation. Scrambled-knockdown C2C12 cells can form proper mutinucleated myotubes. Arrow indicates the striated pattern of MyHC. Arrowheads indicate the striated pattern of myofibrils.
(TIF)

S3 Fig mRNA expression of palladin in control versus knockdown cell lines. (TIF)

S4 Fig Western blots of control versus palladin-depleted cell lines (shPalld-1, shPalld-2). Note that knockdown cell lines displayed elevated MyHC expression at the early stage of differentiation (day 2) versus that of control cells.
(TIF)

S5 Fig qPCR analysis of MEF2C mRNA expression. (TIF)

S1 Table List of primer sequences used for qPCR analysis in this study. (DOCX)
