# Supplementary figures and images for: Dual Roles of Palladin Protein in In Vitro Myogenesis: Inhibition of Early Induction but Promotion of Myotube Maturation
Source: PLoS One. 2015 Apr 14;10(4):e0124762. doi: 10.1371/journal.pone.0124762 (PMC4396843; doi:10.1371/journal.pone.0124762)

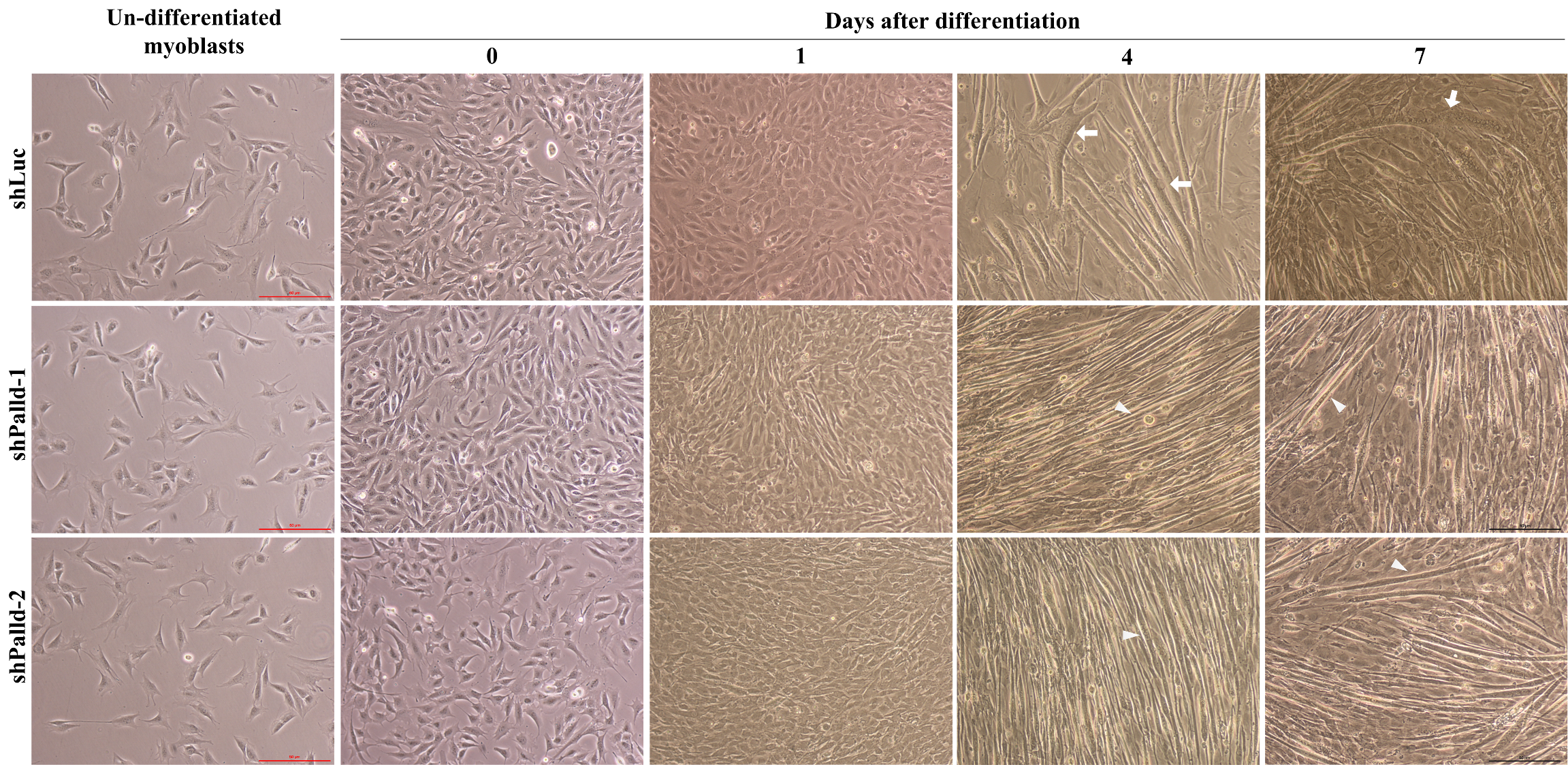

Supplement: S1 Fig — Arrows indicate the mature myotubes. Arrowheads indicate the thin myotube of palladin-knockdown cell lines. (TIF) [file pone.0124762.s001.tif]

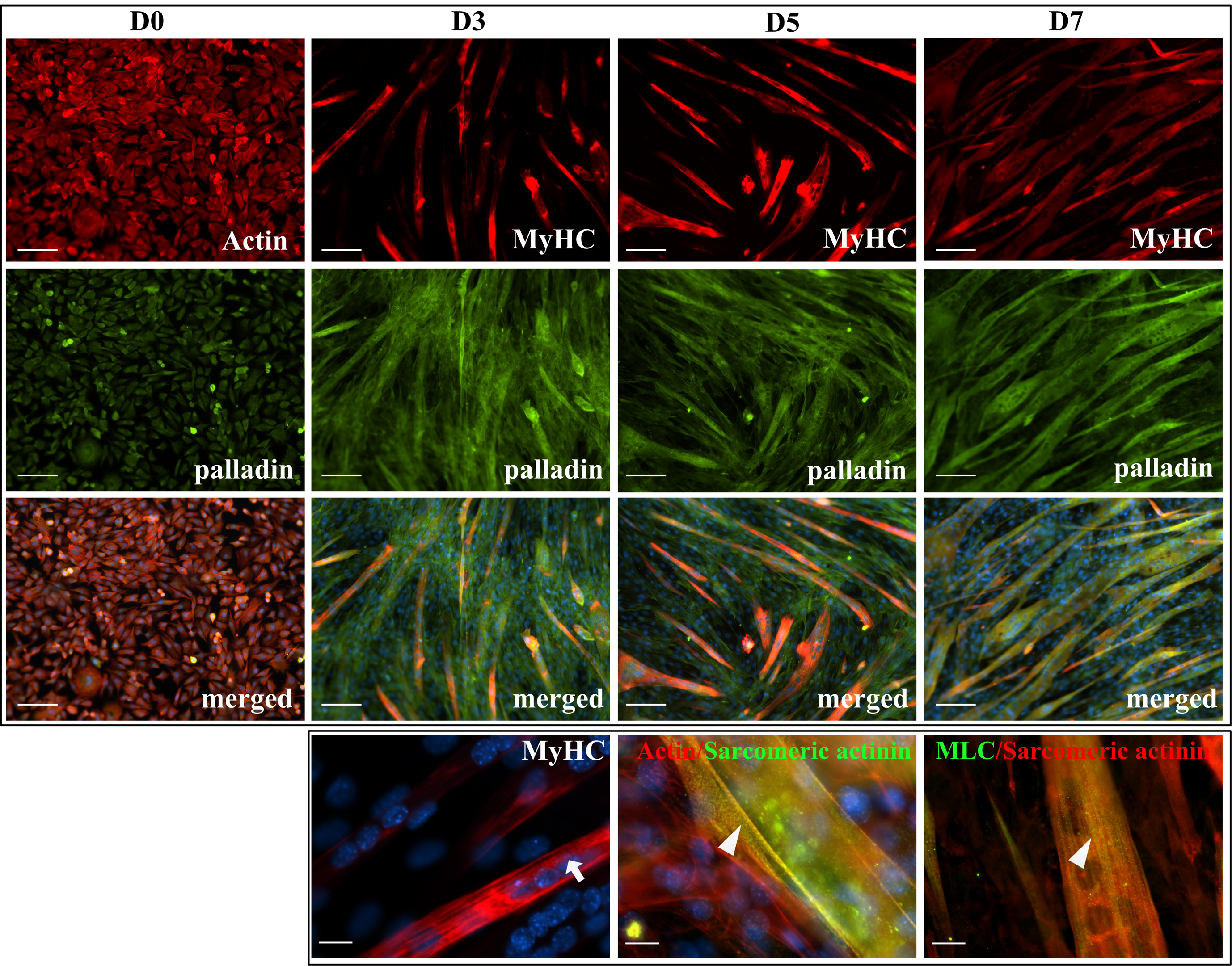

Supplement: S2 Fig — Scrambled-knockdown C2C12 cells can form proper mutinucleated myotubes. Arrow indicates the striated pattern of MyHC. Arrowheads indicate the striated pattern of myofibrils. (TIF) [file pone.0124762.s002.tif]

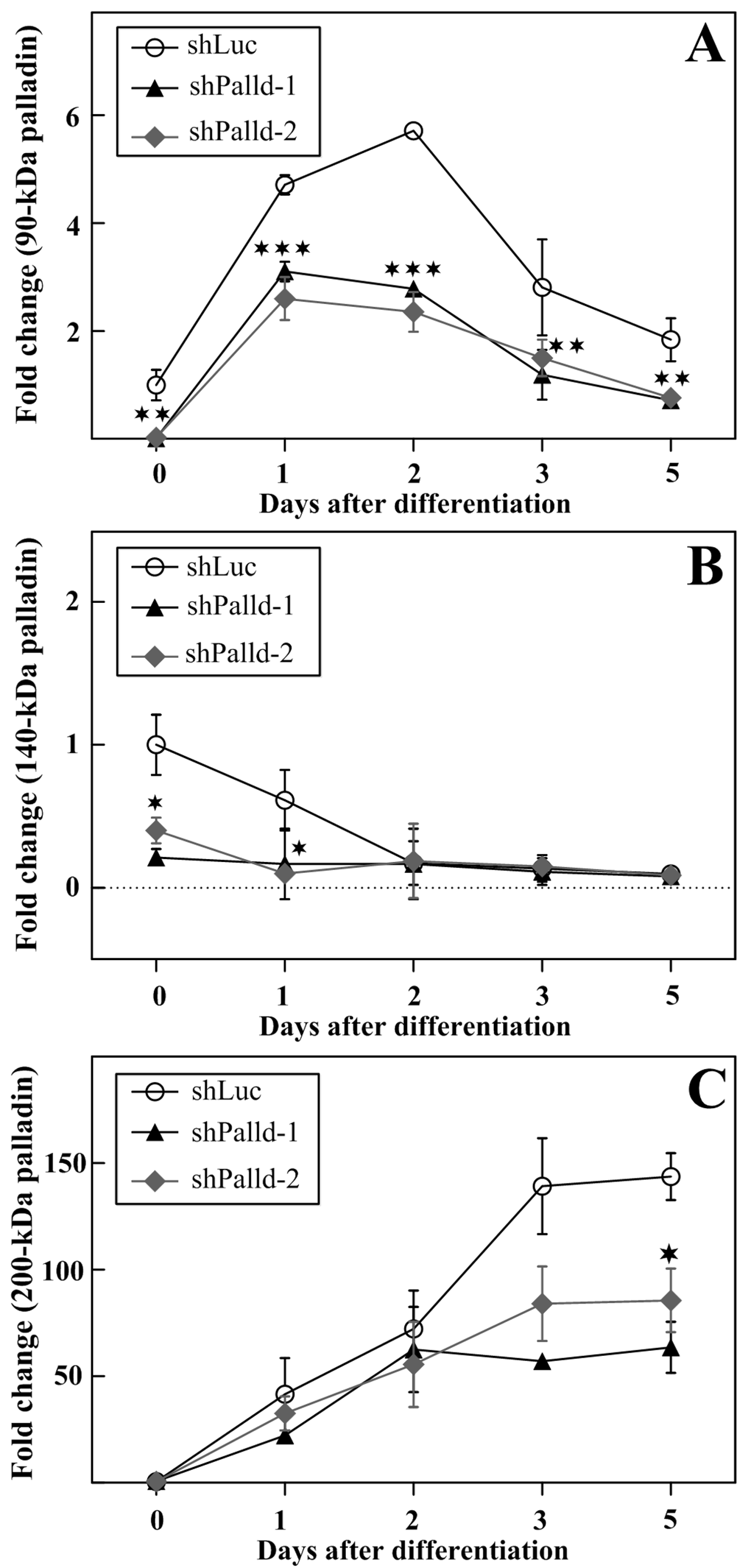

Supplement: S3 Fig — (TIF) [file pone.0124762.s003.tif]

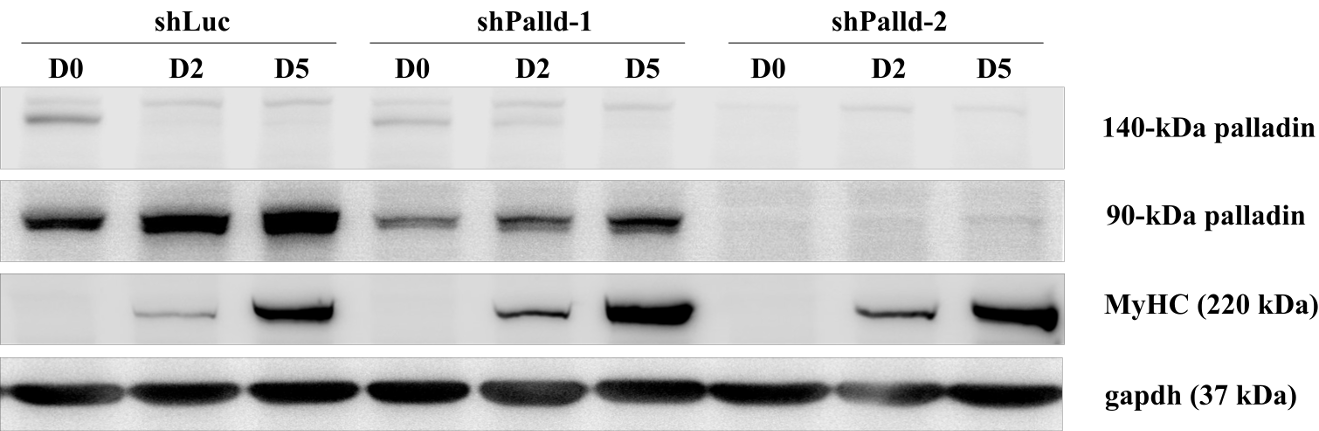

Supplement: S4 Fig — Note that knockdown cell lines displayed elevated MyHC expression at the early stage of differentiation (day 2) versus that of control cells. (TIF) [file pone.0124762.s004.tif]

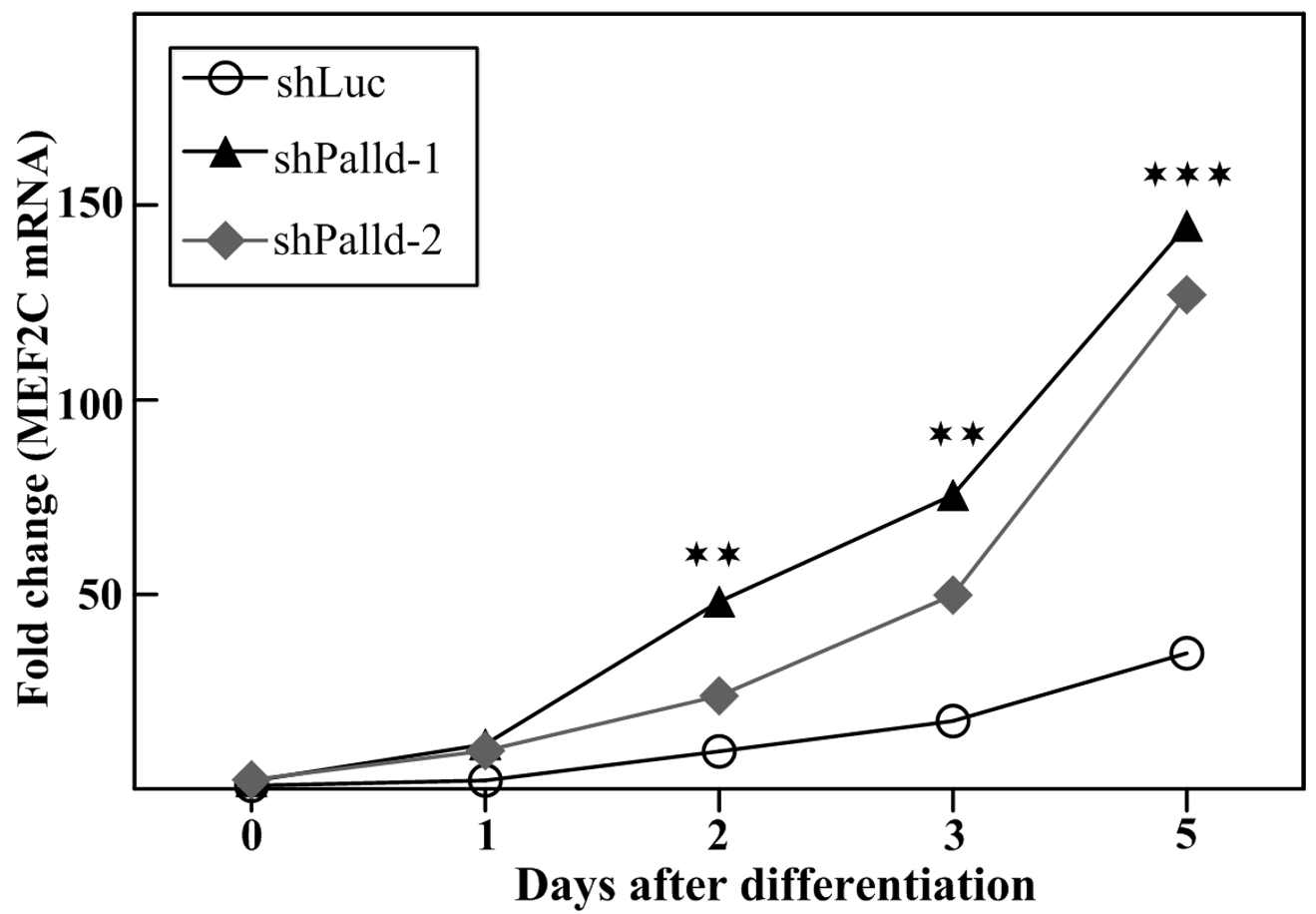

Supplement: S5 Fig — (TIF) [file pone.0124762.s005.tif]
