# Supplementary material for: Dual Roles of Palladin Protein in In Vitro Myogenesis: Inhibition of Early Induction but Promotion of Myotube Maturation
Source: PLoS One. 2015 Apr 14;10(4):e0124762. doi: 10.1371/journal.pone.0124762 (PMC4396843; doi:10.1371/journal.pone.0124762)
Supplement: S1 Table — (DOCX) [file pone.0124762.s006.docx]

**Table S1: List of primer sequences used for qPCR analysis in this study.**

| **Gene** | **Forward primer (5’ to 3’)** | **Reverse primer (5’ to 3’)** |
| --- | --- | --- |
| Igf1 | GGACCAGAGACCCTTTGCGGGG | GGCTGCTTTTGTAGGCTTCAGTGG |
| MEF2C | ATCCCGATGCAGACGATTCAG | AACAGCACACAATCTTTGCCT |
| Myostatin | CAGACCCGTCAAGACTCCTACA | CAGTGCCTGGGCTCATGTCAAG |
| MyHC | AAACCACCTCAGAGTTGTGGA | GTTCCGAAGGTTCCTGATTGC |
| Myogenin | AGTGAATGCAACTCCCACAG | ACGATGGACGTAAGGGAGTG |
| p21 | CCTGGTGATGTCCGACCTG | CCATGAGCGCATCGCAATC |
| 90-kDa palladin | CAGATGGGACTTTTCCGCTC | ACTTGGTTCTGCAGCTGCTG |
| 140-kDa palladin | TGCTGCCTGTGCATTTTCCC | AGCTTTCGCTGTCAGAGTCC |
| 200-kDa palladin | CATCCAGAAACTGAGGAGCC | AGCTTTCGCTGTCAGAGTCC |
| Gapdh | CGACTTCAACAGCAACTCCCACTCTTCC | TGGGTGGTCCAGGGTTTCTTACTCCTT |
